# Association between MRI-based visceral adipose tissues and metabolic abnormality in a Chinese population: a cross-sectional study

**DOI:** 10.1186/s12986-022-00651-x

**Published:** 2022-03-05

**Authors:** Xuhui Zhang, Qiannan Chen, Xiaohui Sun, Qiong Wu, Zongxue Cheng, Qingguo Lv, Jiaqiang Zhou, Yimin Zhu

**Affiliations:** 1 Hangzhou Center for Disease Control and Prevention, Hangzhou, 310051 Zhejiang China; 2 Affiliated Hangzhou Center of Disease Control and Prevention, Zhejiang University School of Public Health, Hangzhou, 310051 Zhejiang China; 3 Basic Discipline of Chinese and Western Integrative, School of Public Health, Zhejiang Chinese Medical University, Hangzhou, 310053 Zhejiang China; 4 Department of Epidemiology and Biostatistics, School of Public Health, Zhejiang Chinese Medical University, Hangzhou, 310053 Zhejiang China; 5 Department of Epidemiology and Biostatistics, School of Public Health, Zhejiang University, Hangzhou, 310058 Zhejiang China; 6 Department of Endocrinology and Metabolism, West China Hospital, Sichuan University, Chengdu, 610000 Sichuan Province China; 7 Department of Endocrinology, Sir Run Run Shaw Hospital Affiliated to School of Medicine, Zhejiang University, Hangzhou, 310058 Zhejiang China; 8 Department of Respiratory Diseases, Sir Run Run Shaw Hospital Affiliated to School of Medicine, Zhejiang University, Hangzhou, 310058 Zhejiang China; 9 Department of Pathology, School of Medicine, Zhejiang University, Hangzhou, 310058 Zhejiang China

**Keywords:** Obesity, Subcutaneous adipose tissue (SAT), Visceral adipose tissue (VAT), Body mass index (BMI), Metabolic abnormality (MA)

## Abstract

**Background:**

Previous studies have indicated that the deposition of abdominal adipose tissue was associated with the abnormalities of cardiometabolic components. The aim of this study was to examine the relationship of visceral adipose tissue (VAT), subcutaneous adipose tissue (SAT) and metabolic status and the different effects between males and females.

**Methods:**

The 1388 eligible subjects were recruited in a baseline survey of metabolic syndrome in China, from two communities in Hangzhou and Chengdu. Areas of abdominal VAT and SAT were measured by magnetic resonance imaging (MRI). Serum total triglycerides (TG), high-density lipoprotein cholesterol (HDL-C) were measured by an automated biochemical analyzer. Metabolic abnormality (MA) was defined more than one abnormal metabolic components, which was based on the definition of metabolic syndrome (IDF 2005). Multiple logistic regression was used to calculate the odds ratios (ORs) and 95% confidence intervals (95%CI). Predictive value was assessed by area under the curve (AUC), net reclassification improvement (NRI), and integrated discrimination improvement (IDI), respectively.

**Results:**

Their mean age was 53.8 years (SD: 7.1 years), the mean body mass index (BMI) was 23.7 kg/m^2^, and 44.8% of the subjects were male. Both male and female with MA had higher VAT levels compared to subjects with normal metabolism (MN), and male had higher SAT levels than female (*P* < 0.05). Higher VAT was significantly associated with MA with ORs in the fourth quartile (Q4) of 6.537 (95% CI = 3.394–12.591) for male and 3.364 (95% CI = 1.898–5.962) for female (*P* for trend < 0.05). In female, VAT could increase the risk of metabolic abnormalities, but SAT could increase the risk of MA in the second and fourth quartiles (Q2 and Q4) only at BMI > 24 kg/m^2^. In male, VAT improved the predictive value of MA compared to BMI and waist circumference (WC), the AUC was 0.727 (95% CI = 0.687–0.767), the NRI was 0.139 (95% CI = 0.070–0.208) and 0.106 (95% CI = 0.038–0.173), and the IDI was 0.074 (95% CI = 0.053–0.095) and 0.046 (95% CI = 0.026–0.066). Similar results were found in female.

**Conclusions:**

In male, VAT and SAT could increase the risk of metabolic abnormalities both at BMI < 24 kg/m^2^ and at BMI ≥ 24 kg/m^2^. In female, VAT could increase the risk of metabolic abnormalities but SAT could increase the risk of MA in the second and fourth quartiles (Q2 and Q4) only at BMI > 24 kg/m^2^. Deposition of abdominal adipose tissue was associated with metabolic abnormalities. VAT improved the predictive power of MA.

**Supplementary Information:**

The online version contains supplementary material available at 10.1186/s12986-022-00651-x.

## Introduction

Obesity, especially central obesity, is a well-established risk factor for a several diseases, such as dyslipidemia, type 2 diabetes (T2DM), cardiovascular diseases (CVD), and all-cause mortality [1, 2]. Body mass index (BMI) and waist circumference (WC) were widely used to evaluate obesity. However, BMI does not fully characterize adiposity, which is limited by age, sex and race specific BMI in body fat [3]. Although WC reflected central obesity and is readily available, it does not adequately reflect actual body adipose tissue distribution and therefore fail to distinguish between abdominal subcutaneous adipose tissue (SAT) and visceral adipose tissue (VAT), and SAT and VAT have different metabolic consequences [4]. Studies on the effects of SAT on metabolic abnormality are still inconclusive and even contradictive [1, 5–7]. Some studies have found SAT to be a beneficial fat depot for type 2 diabetes and metabolic syndrome [7, 8], however, others have not found a significant correlation between SAT and some components of metabolic abnormalities [5, 6, 9]. In addition, because of sex-difference in fat accumulation between male and female, the extent to which it affects metabolic abnormalities across sexes is unclear. Some studies have found a causal relationship between higher VAT and cardiometabolic risk factors, with a greater effect on female [10]. While other studies indicated that the absolute risk contribution of VAT to metabolic factors is greater in male than in female [11], and various findings also emphasize the sex differences in regional fat distribution.

One possible reason for these sophisticated associations is the different fat distribution in different ethnic groups [12, 13]. In addition, the way in which SAT and VAT are measured and the adjustment for confounding factors are also possible causes. Currently, techniques to accurately assess regional adipose depots include computed tomography (CT) and magnetic resonance imaging (MRI) and so on [14]. MRI-based adipose tissue measurements that can directly quantify abdominal fat compartments and without radiation. In the Chinese population, limited studies have explored the effects of MRI-measured SAT, VAT on metabolic disorders by sex. Therefore, we examined the relationship between SAT, VAT and metabolic abnormality in male and female separately.

## Materials and methods

### Subjects

These subjects were recruited in a baseline investigation of metabolic syndrome investigation in China in 2010. The participants were recruited if they were ≥ 18 years old, detailed information has been described in our previous study [15]. In this study, a subpopulation from two communities in Hangzhou (n = 1170) and Chengdu (n = 761) was included. Subjects were excluded if they had (1) severe chronic diseases including cardiovascular diseases (including angina, cerebral infarction), cancers, renal dysfunction and other chronic wasting diseases and (2) missing anthropometric information, SAT and VAT data. A total of 1388 eligible subjects were eventually included.

This study was approved by the institutional Review Board of Zhejiang University, China. All participants provided their written informed consents.

### SAT and VAT measurements

Abdominal adipose tissue was measured by magnetic resonance imaging (MRI) using a whole-body imaging system (SMT-100, Shimadzu, Japan) with TR-500 and TE-200 of SE. MRI scans were performed at the interface of the umbilicus (approximately the lower edge of L4) with the subject in the supine position. The area of subcutaneous and visceral adipose tissue was calculated using sliceOmatic software (version 4.2).

### Covariant assessment

With a standard questionnaire, the information including age, sex, smoking (current, former, and never), alcohol drinking behaviors (never, moderate, and heavy), menstrual history (for female) and disease history were collected. Current smoking was defined as smoking at least one cigarette per day and lasting for one year. Previous smoking was considered to have quit smoking for at least one year. They were classified as heavy drinkers, moderate drinkers and never drinkers according to the frequency of alcohol consumption: more than 3 times a week was classified as heavy drinking. A local nurse or investigator asked the subjects if they have any diseases such as hypertension and if they use medication. Anthropometric variables were collected by trained investigators following a standard protocol [15] and included weight, height, waist circumference (WC), systolic blood pressure (SBP), and diastolic blood pressure (DBP). The protocols were briefly described below. BMI was calculated as weight (kg) divided by the square of height (m). Height and weight were measured when the subjects wore light clothing and without shoes. WC was measured at the midpoint between the iliac crest and lowest rib. Blood pressure was investigated in a seated position with a mercury sphygmomanometer. SBP and DBP were measured as the average of three repeat measurements with an interval of at least 30 s.

The overnight fasting blood samples were collected for each subject. Total triglycerides (TG), total cholesterol (TC), high-density lipoprotein cholesterol (HDL-C), low-density lipoprotein cholesterol (LDL-C) were measured by biochemical auto-analyzers (Hitachi 7060, Tokyo, Japan). Fasting plasma glucose (FPG) was analyzed using the glucose oxidase method with a Beckman Glucose Analyzer (Beckman Instruments, Irvine, CA, USA). A 2 h oral glucose tolerance test (OGTT-2h) was performed as a routine procedure for the subjects, except for patients with previously diagnosed diabetes.

The metabolic abnormality component was defined according to the 2005 International Diabetes Federation (IDF) criteria for metabolic syndrome [16], including elevated TG ≥ 1.7 mmol/L, low HDL-C < 1.03 mmol/L (in male), < 1.29 mmol/L (in female); elevated FPG ≥ 5.6 mmol/L or a history of diabetes, or used antidiabetic drugs; elevated SBP ≥ 130 mmHg, or DBP ≥ 85 mmHg or used antihypertensive drugs. Metabolic abnormality (MA) was defined as more than one abnormal metabolic component, and metabolic normality (MN) was defined as zero or only one abnormal metabolic component [15]. According to the BMI standard for Chinese adults proposed by the China Working Group on Obesity (WGOC), a BMI threshold of 24 kg/m^2^ [17] was used to explore the correlation between SAT, VAT and MA in different Chinese populations at normal and abnormal BMI.

### Statistical analysis

Continuous variables are presented as mean ± standard deviation (SD) or median and inter-quartile range (IQR). Categorical variables were shown as numbers (%). Student’s t-test or the Wilcoxon rank-sum test was used to compare continuous variables. Chi-square tests were used to compare categorical variables. The subjects were divided into four groups by quartiles of SAT and VAT, with the first quartile (Q1) as the reference group. The ORs and 95%CIs for each quartile using multiple logistic regression, adjusted for age, BMI (for overall), smoke, drink, menstrual history (for female). A two-tailed *P* < 0.05 was considered statistically significant. Two packages of “Predict ABEL” and “pROC” were used to calculate the net reclassification improvement (NRI), integrated discrimination improvement (IDI), area under curve (AUC) and so on. The software IBM SPSS Statistics version 25.0 and R 3.6.3 were used to analyze the data.

## Results

### The baseline characteristics of subjects

The baseline characteristics by sex are summarized in Table 1. Of the 1388 subjects, 622 (44.8%) were male, 766 (55.2%) were female. Their mean age was 53.8 years (SD = 7.1). The median VAT for male was 91.0 cm^2^ (55.1–127.4 cm^2^) higher than 60.4 cm^2^ (43.3–79.6 cm^2^) for female (*P* < 0.05), and the median SAT for male was 123.2 cm^2^ (98.1–149.8 cm^2^) lower than 178.2 cm^2^ (139.1–221.6 cm^2^) for female (*P* < 0.05). Compared to female, male had higher BMI, WC, WHR, VAT, SBP, DBP, FPG, OGTT-2 h, TG, and higher prevalence of metabolic abnormality; however, female had higher TC and HDL-C (all the *P* values < 0.05).

**Table 1:** Characteristics of the subjects stratified by sex

Characteristics	Total (n = 1388)	Male (n = 622)	Female (n = 766)	*P*
Age (years)	53.8 ± 7.1	53.6 ± 7.1	53.9 ± 7.1	0.539
BMI (kg/m^2^)	23.70 ± 2.99	24.18 ± 2.99	23.31 ± 2.93	< 0.001
WC (cm)	79.3 ± 9.0	83.3 ± 8.4	76.0 ± 8.1	< 0.001
WHR	0.87 ± 0.07	0.90 ± 0.06	0.84 ± 0.06	< 0.001
SAT area (cm^2^)	148.5 (112.9–194.7)	123.2 (98.1–149.8)	178.2 (139.1–221.6)	< 0.001
VAT area (cm^2^)	69.5 (45.5–107.2)	91.0 (55.1–127.4)	60.4 (43.3–79.6)	< 0.001
SBP (mmHg)	120.5 ± 15.8	123.7 ± 15.4	117.8 ± 15.6	< 0.001
DBP (mmHg)	79.5 ± 9.8	82.4 ± 9.8	77.2 ± 9.2	< 0.001
FPG (mmol/L)	5.12 ± 1.17	5.23 ± 1.41	5.03 ± 0.92	0.003
OGTT-2 h (mmol/L)	6.65 ± 3.30	6.97 ± 3.93	6.38 ± 2.65	0.002
TC (mmol/L)	5.28 ± 1.08	5.13 ± 1.00	5.40 ± 1.13	< 0.001
TG (mmol/L)	1.30 (0.90–1.87)	1.47 (1.00–2.19)	1.20 (0.85–1.70)	< 0.001
HDL-C (mmol/L)	1.48 ± 0.36	1.36 ± 0.33	1.58 ± 0.36	< 0.001
LDL-C (mmol/L)	2.59 ± 0.67	2.57 ± 0.67	2.61 ± 0.66	0.273
MA (n, %)	667 (48.1%)	357 (57.4)	310 (40.5)	< 0.001

Data are presented as means ± standard deviation or medians (inter-quartile ranges) or n (percentage). *BMI* body mass index, *WC* waist circumference, *WHR* waist-to-hip ratio, *SAT* subcutaneous adipose tissue, *VAT* visceral adipose tissue, *SBP* systolic blood pressure, *DBP* diastolic blood pressure, *FPG* fasting plasma glucose, *OGTT-2h* 2 h post oral glucose tolerance test, *TC* total cholesterol, *TG* triglyceride, *HDL-C* high density lipoprotein cholesterol, *LDL-C* low density lipoprotein cholesterol, *MA* metabolic abnormality, which was defined as metabolic abnormal components ≥ 2, which were based on the definition of metabolic syndrome (IDF 2005)

### Levels of SAT, VAT in different metabolic status stratified by sex and BMI

The levels of SAT and VAT in metabolic abnormalities stratified by sex and BMI are presented in Table 2. In male, the median SAT for MA was 130.6 cm^2^ (106.2–159.5 cm^2^) higher than 110.8 cm^2^ (80.3–141.3 cm^2^) for MN group. The median VAT for MA was 110.5 cm^2^ (75.2–136.8 cm^2^) higher than 64.5 cm^2^ (32.1–101.2 cm^2^) for MN group (all the *P* values < 0.05). Similar results were found in female (Table 2).

**Table 2:** Levels of SAT, VAT in different metabolic status stratified by sex and BMI

	Male			Female		
	MN	MA	*P*	MN	MA	*P*
Overall						
SAT	110.8 (80.3–141.3)	130.6 (106.2–159.5)	< 0.001	170.3 (133.9–205.9)	191.0 (148.8–239.3)	< 0.001
VAT	64.5 (32.1–101.2)	110.5 (75.2–136.8)	< 0.001	53.4 (36.0–68.8)	75.2 (55.9–107.2)	< 0.001
BMI < 24 kg/m^2^						
SAT	90.7 (66.92–112.2)	106.5 (82.7–121.4)	0.001	154.5 (125.9–183.1)	155.2 (126.7–192.6)	0.378
VAT	42.1 (22.8–67.8)	77.10 (53.90–104.05)	< 0.001	48.9 (34.2–62.6)	59.9 (46.6–78.3)	< 0.001
BMI ≥ 24 kg/m^2^						
SAT	139.5 (116.1–161.7)	148.0 (125.6–180.9)	0.004	212.5 (180.8–250.6)	223.6 (184.3–265.0)	0.066
VAT	97.8 (69.4–122.9)	126.6 (101.7–155.8)	< 0.001	66.4 (47.0–92.7)	94.0 (68.0–123.1)	< 0.001

Data are presented as medians (inter-quartile ranges)

*BMI* body mass index, *MN* metabolic normality, which was defined as abnormally metabolic components ≤ 1, *MA* metabolic abnormality, which was defined as metabolic abnormal components ≥ 2

When stratified by levels of BMI at 24 kg/m^2^, subjects with MA had significantly higher levels of SAT and VAT than MN group in male. In female, only VAT was relatively high in MA, and the difference was statistically significant (*P* < 0.05).

### The associations of different levels of SAT, VAT with metabolic abnormality stratified by sex and BMI

Table 3 shows the associations of SAT, VAT with metabolic abnormality stratified by sex and BMI after adjusted for age, BMI (for overall), smoke, drink, and menstrual history (for female). In male and female, VAT was significantly correlated with the higher risk of MA (*P* for trend < 0.05). Compared with the reference group for the first quartile (Q1), the ORs in fourth quartile (Q4) were 6.537 (95% CI = 3.394–12.591) for male and 3.364 (95% CI = 1.898–5.962) for female, respectively. However, there was no association between SAT and MA when BMI was not grouped. Since there are relatively few Q3 and Q4 males with metabolic abnormality when BMI < 24 kg/m^2^, so we put Q3 and Q4 males together for analysis.

**Table 3:** The relationships between SAT, VAT and metabolic abnormality stratified by sex and BMI

	Male			Female		
	n	%	OR (95%CI)	n	%	OR (95%CI)
Overall						
SAT						
Q1	60	38.7	ref	60	31.4	Ref
Q2	91	58.7	1.458 (0.878–2.421)	70	36.6	0.833 (0.518–1.338)
Q3	97	62.6	1.344 (0.762–2.371)	76	39.4	0.667 (0.403–1.103)
Q4	109	70.3	1.391 (0.707–2.735)	104	54.5	0.576 (0.319–1.040)
* P* for trend			0.445			0.05
VAT						
Q1	45	29	ref	40	20.9	ref
Q2	85	54.5	2.530 (1.512–4.232)	64	33.3	1.495 (0.914–2.444)
Q3	105	67.3	3.939 (2.199–7.053)	77	40.1	1.565 (0.946–2.589)
Q4	122	78.7	6.537 (3.394–12.591)	129	67.5	3.364 (1.898–5.962)
* P* for trend			< 0.001			< 0.001
BMI < 24 kg/m^2^						
SAT						
Q1	50	38.8	ref	47	30.1	ref
Q2	54	56.8	2.062 (1.177–3.613)	49	30.8	0.911 (0.549–1.511)
Q3 and Q4	32	56.1	2.121 (1.103–4.078)	31	29.5	0.896 (0.510–1.575)
Q4				20	47.6	1.631 (0.781–3.407)
* P* for trend			0.009			0.463
VAT						
Q1	36	28.1	ref	29	19.5	Ref
Q2	52	59.1	3.505 (1.945–6.314)	46	31.1	1.631 (0.936–2.845)
Q3 and Q4	48	71.6	6.026 (3.079–11.795)	40	33.6	1.770 (0.988–3.168)
Q4				32	69.6	7.422 (3.422–16.095)
* P* for trend			< 0.001			< 0.001
BMI ≥ 24 kg/m^2^						
SAT						
Q1	10	38.5	ref	13	37.1	Ref
Q2	37	61.7	2.516 (0.949–6.672)	21	65.6	4.753 (1.531–14.755)
Q3	70	64.8	2.823 (1.132–7.039)	45	51.1	2.474 (0.968–6.323)
Q4	104	71.7	3.862 (1.573–9.484)	84	56.4	2.502 (1.021–6.129)
* P* for trend			0.005			0.350
VAT						
Q1	9	33.3	ref	11	26.2	ref
Q2	33	48.5	1.703 (0.656–4.420)	18	40.9	2.185 (0.803–5.944)
Q3	71	64	3.244 (1.305–8.064)	37	50.7	2.576 (1.024–6.478)
Q4	108	81.2	7.836 (3.086–19.893)	97	66.9	4.607 (1.909–11.118)
* P* for trend			< 0.001			< 0.001

Data are presented as OR (95%CI). The "n" was the case of MA, and "%" means the proportion of MA in the subgroups

*BMI* body mass index. The ORs was adjusted for age, BMI (for overall), smoke, drink, and menstrual history (for female). Male: SAT:Q1 (< 98.1), Q2 (98.1−), Q3 (123.2−), Q4 (149.8−); VAT:Q1 (< 55.1), Q2 (55.1−), Q3 (91.00−), Q4 (127.4−); Female:SAT:Q1 (< 139.1), Q2 (139.1−),Q3 (178.2−),Q4 (221.6−); VAT:Q1 (< 43.0), Q2 (43.0−), Q3 (60.4−), Q4 (79.6−)

When stratified by BMI level of 24 kg/m^2^, VAT was found to be significantly associated with MA in both male and female. However, for SAT, different effects were found between males and females. In male, SAT were consistently associated with the risk of MA for both BMI < 24 kg/m^2^ and BMI ≥ 24 kg/m^2^ (*P* for trend < 0.05). In female, SAT could increase the risk of MA only when BMI ≥ 24 kg/m^2^. Additional File 1: Table S2 show the relationship between SAT, VAT and metabolic components, indicating that SAT may be a protective factor for high BS (blood sugar) in female, with an OR for Q4 was 0.383 (0.185–0.792) (*P* for trend < 0.05).

### The predictive abilities of VAT and SAT for metabolic abnormality

Table 4 describes the predictive abilities of VAT and SAT for metabolic abnormality. In male, the AUC of VAT was 0.727 (95%CI = 0.687–0.767), significantly higher than BMI (0.658, 95%CI = 0.614–0.701) and WC (0.688, 95%CI = 0.646–0.730) (all the *P* values < 0.05). VAT could improve the predictive value of MA compared with BMI or WC, with NRIs (95%CI) of 0.139 (0.070, 0.208) and 0.106 (0.038, 0.173), respectively; and the IDIs (95%CI) were 0.074 (0.053, 0.095) and 0.046 (0.026, 0.066), respectively. But SAT was less predictive of metabolic abnormalities than WC and BMI.

**Table 4:** The predictive values on metabolic abnormality in BMI, WC, SAT and VAT

	AUC (95%CI)	*Z*	*P*	NRI (95%CI) *	*P*	IDI (95%CI)*	*P*
Male							
BMI	0.658 (0.614–0.701)						
WC	0.688 (0.646–0.730)						
SAT	0.639 (0.594–0.683)	1.160	0.246	0.070 (− 0.004, 0.144)	0.062	− 0.022 (− 0.036, − 0.007)	0.003
SAT*		3.012	0.003	− 0.029 (− 0.102, 0.044)	0.442	− 0.050 (− 0.066, − 0.034)	< 0.001
VAT	0.727 (0.687–0.767)	− 3.864	< 0.001	0.139 (0.070, 0.208)	< 0.001	0.074 (0.053, 0.095)	< 0.001
VAT*		− 2.458	0.014	0.106 (0.038, 0.173)	0.003	0.046 (0.026, 0.066)	< 0.001
Female							
BMI	0.666 (0.627–0.705)						
WC	0.693 (0.655–0.732)						
SAT	0.602 (0.560–0.643)	3.895	< 0.001	− 0.057 (− 0.125, 0.012)	0.106	− 0.052 (− 0.067, − 0.037)	< 0.001
SAT*		5.095	< 0.001	− 0.118 (− 0.188, − 0.048)	0.001	− 0.074 (− 0.092, − 0.056)	< 0.001
VAT	0.712 (0.674–0.749)	− 2.562	0.010	0.112 (0.037, 0.188)	0.004	0.050 (0.028, 0.072)	< 0.001
VAT*		− 1.115	0.265	0.042 (− 0.031, 0.114)	0.261	0.028 (0.007, 0.049)	0.008

*The predictive values in VAT and SAT compared to WC

*AUC* area under curve, *NRI* net reclassification improvement, *IDI* integrated discrimination improvement

Similar results were found in female (Table 4), with an AUC of 0.712 (95%CI = 0.674–0.749) for VAT, significantly higher than BMI (0.666, 95%CI = 0.627–0.705) and WC (0.693, 95%CI = 0.655–0.732) (all the *P* values < 0.05). Compared with BMI and WC, VAT improved the predictive value.

## Discussion

In this cross-sectional study, we found that higher VAT, but not SAT, was associated with the risk of MA when BMI was used as a covariate. However, after BMI stratification, SAT and VAT in men could increase the risk of MA at all levels of BMI. For women, SAT could increase the risk of MA in the second and fourth quartiles (Q2 and Q4) only at BMI > 24 kg/m^2^. Compared with BMI and WC, VAT improved the predictive power of MA. Deposition of abdominal adipose tissue was associated with the risk of MA.

In fact, there are some differences between SAT and VAT in anatomy, cytology, molecular, physiology, clinical and so on [18]. The VAT is considered to be the more pathogenic adipose tissue compartment compared to the SAT [19]. This may be related to the biological function of VAT, a metabolically active organ that includes more non-adipocytes, including macrophages, immune cells, preadipocytes and fibroblasts, and can secrete amounts of inflammation mediators to induce metabolic disorders [18, 20–22]. And in our Additional file 1: Table S1, We found that VAT was positively associated with both high TG and low HDL-C. In addition, the high lipolytic activity of VAT and its accompanying inflammatory response also contribute to abnormal lipogenesis, glucose homeostasis, and vascular health [23, 24]. Thus, a higher VAT may increase the risk of developing metabolic abnormalities. With regards to the contribution of VAT in different sex, inconclusive results were reported [10, 11, 25–27]. Several Caucasian studies have shown that VAT is more strongly associated with type 2 diabetes, hypertension and hyperlipidemia in female [10, 25, 28]. In our Additional file 1: Table S3, we observed that the effect of VAT on high TG and low-HDL was higher in male, indicating that VAT may have more striking effect on lipid metabolism in male than female. The possible reason maybe that only a limited number of confounders were adjusted, which may have affected the results. An expanded study of the Chinese population is necessary to determine the gender differences in the contribution of VAT. In general, the relationship between VAT and metabolic outcomes is relatively stable, which may be related to multiple biological effects of VAT.

SAT is known to have adverse effects on a variety of metabolic risk factors and may have unique pathogenic properties independent of BMI [1, 6, 25, 29], and the effects of different levels of SAT on cardiometabolic factors are inconsistent [1, 6, 13, 19, 25, 30]. Consistent with previous studies [30–32], our study (See Additional file 1: Tables S1, S2) showed that higher SAT was not associated with hypertension, higher TG, and lower HDL-C risk after adjustment for age, smoke, drink, and menstrual history (for women), and SAT may be a protective factor for blood sugar. Several studies with European or African populations have found independent associations of SAT with high blood pressure (H-BP) and HDL-C [1, 14, 26], suggesting that SAT has different effects in different ethnic groups. A possible explanation for this sex difference in SAT is the different sex steroid hormone profiles, as these sex hormones are important in regulating adipose tissue distribution and energy metabolism [33, 34]. There are also several hypotheses for the protective effect of SAT to explain this observation. One is that smaller adipocytes, SAT are more sensitive to insulin and have a greater capacity to absorb fatty acids and triglycerides and therefore can act as a powerful buffer to prevent excess fat from entering non-adipose tissue [35]. On the other hand, SAT can secrete more favorable adipokines such as adiponectin, with antidiabetics and antiatherogenic properties [18, 23]. Therefore, the different effects of SAT on metabolic outcomes may be related to its biological functions. Since SAT has different effects on metabolic components in different sexes, it may result in a less stable correlation between SAT and metabolic abnormality.

Previous studies have shown that baseline and changes in VAT were independent predictors of future dyslipidemia, but BMI and SAT were not associated with future development of atherosclerotic dyslipidemia [36]. This result is consistent to our study that VAT is a better predictor for MA compared with BMI and WC.

There are some advantages in our study. Areas of SAT and VAT were measured using MRI, which is the gold standard method of determining abdominal adipose tissue [37]. The data, including anthropometric and questionnaire-based information, were collected by trained health professionals, and the biochemical measurements followed the standard protocols. Our study also has some limitations. First, we cannot infer a causal relationship between the adipose indices and the metabolic abnormality because of the cross-sectional design. Second, this study included limited confounding factors, such as not including regional fat distribution, such as deep SAT and superficial SAT, and medication use, which may have biased the results. Thirdly, the sample size of this study was relatively small. Finally, our data were based on only one single ethnic group, thus the results may not be applied to other ethnicities.

## Conclusions

In male, VAT and SAT could increase the risk of metabolic abnormalities both at BMI < 24 kg/m^2^ and at BMI ≥ 24 kg/m^2^. In female, VAT could increase the risk of metabolic abnormalities but SAT could increase the risk of MA in the second and fourth quartiles (Q2 and Q4) only at BMI > 24 kg/m^2^. Deposition of abdominal adipose tissue was associated with metabolic abnormalities. VAT improved the predictive power of MA.

## Supplementary Information

**Additional file 1.** Supplementary Tables.

## Data Availability

The datasets used and/or analyzed during the current study are available from the corresponding author on reasonable request.

## Abbreviations

SAT: Subcutaneous adipose tissue
VAT: Visceral adipose tissue
MRI: Nuclear magnetic resonance imaging
BMI: Body mass index
WC: Waist circumference
WHR: Waist-to-hip ratio
SBP: Systolic blood pressure
DBP: Diastolic blood pressure
FPG: Fasting plasma glucose
OGTT-2h: 2-Hour post oral glucose tolerance test
TC: Total cholesterol
TG: Triglyceride
HDL-C: High density lipoprotein cholesterol
LDL-C: Low density lipoprotein cholesterol
MA: Metabolic abnormality
MN: Metabolic normality
