# Supplementary material for: Association between MRI-based visceral adipose tissues and metabolic abnormality in a Chinese population: a cross-sectional study
Source: Nutr Metab (Lond). 2022 Mar 5;19:16. doi: 10.1186/s12986-022-00651-x (PMC8898486; doi:10.1186/s12986-022-00651-x)
Supplement: Supplementary file 1 — Additional file 1. Supplementary Tables. [file 12986_2022_651_MOESM1_ESM.docx]

**Additional file 1**

| Supplemental Table 1. The relationships between levels of SAT, VAT and high-TG, low-HDL stratified by sex and BMI | | | | | | | | | | | | | | | |
| --- | --- | --- | --- | --- | --- | --- | --- | --- | --- | --- | --- | --- | --- | --- | --- |
|  | High-TG (male) | | |  | High-TG (female) | | |  | Low-HDL(male) | | |  | Low-HDL(female) | | |
|  | n | % | OR (95%CI) |  | n | % | OR (95%CI) |  | n | % | OR (95%CI) |  | n | % | OR (95%CI) |
| Overall |  |  |  |  |  |  |  |  |  |  |  |  |  |  |  |
| SAT |  |  |  |  |  |  |  |  |  |  |  |  |  |  |  |
| Q1 | 40 | 25.8 | 1(Reference) |  | 38 | 19.9 | 1(Reference) |  | 16 | 10.3 | 1(Reference) |  | 32 | 16.8 | 1(Reference) |
| Q2 | 68 | 43.9 | **1.783(1.062-2.994)** |  | 45 | 23.6 | 1.003(0.600-1.678) |  | 25 | 16.1 | 1.458(0.713-2.981) |  | 41 | 21.5 | 1.124(0.655-1.931) |
| Q3 | 73 | 47.1 | **1.765(1.000-3.115)** |  | 54 | 28.0 | 1.032(0.607-1.755) |  | 32 | 20.6 | 1.825(0.860-3.872) |  | 42 | 21.8 | **0.924(0.523-1.633)** |
| Q4 | 80 | 51.6 | 1.691(0.867-3.297) |  | 60 | 31.4 | 0.789(0.423-1.472) |  | 38 | 24.5 | 1.884(0.794-4.475) |  | 39 | 20.4 | **0.624(0.312-1.250)** |
| P for trend |  |  | 0.203 |  |  |  | 0.513 |  |  |  | 0.151 |  |  |  | 0.160 |
| VAT |  |  |  |  |  |  |  |  |  |  |  |  |  |  |  |
| Q1 | 29 | 18.7 | 1(Reference) |  | 23 | 12.0 | 1(Reference) |  | 10 | 6.5 | 1(Reference) |  | 24 | 12.6 | 1(Reference) |
| Q2 | 59 | 37.8 | **2.737(1.574-4.759)** |  | 40 | 20.8 | 1.549(0.873-2.747) |  | 21 | 13.5 | **2.502(1.090-5.743)** |  | 36 | 18.8 | 1.633(0.913-2.921) |
| Q3 | 80 | 51.3 | **4.786(2.623-8.733)** |  | 60 | 31.3 | **2.601(1.476-4.583)** |  | 37 | 23.7 | **5.218(2.238-12.168)** |  | 40 | 20.8 | **1.828(1.007-3.316)** |
| Q4 | 94 | 60.6 | **6.974(3.628-13.406)** |  | 74 | 38.7 | **3.072(1.627-5.800)** |  | 43 | 27.7 | **6.907(2.829-16.862)** |  | 54 | 28.3 | **2.669(1.371-5.195)** |
| P for trend |  |  | **<0.001** |  |  |  | **<0.001** |  |  |  | **<0.001** |  |  |  | **0.005** |
| BMI＜24kg/m2 |  |  |  |  |  |  |  |  |  |  |  |  |  |  |  |
| SAT |  |  |  |  |  |  |  |  |  |  |  |  |  |  |  |
| Q1 | 33 | 25.6 |  |  | 33 | 21.2 |  |  | 12 | 9.3 |  |  | 27 | 17.3 |  |
| Q2 | 38 | 40.0 | 1.895(0.964-3.726) |  | 31 | 19.5 | 0.784(0.436-1.409) |  | 14 | 14.7 | 1.004(0.393-2.562) |  | 28 | 17.6 | 0.851(0.456-1.586) |
| Q3 | 20 | 42.6 | 2.163(0.915-5.112) |  | 25 | 23.8 | 0.926(0.481-1.783) |  | 11 | 23.4 | 1.541(0.532-4.460) |  | 18 | 17.1 | 0.710(0.347-1.450) |
| Q4 | 6 | 60.0 | **4.234(1.039-17.259)** |  | 17 | 40.5 | 1.602(0.688-3.731) |  | 1 | 10.0 | 0.556(0.061-5.098) |  | 9 | 21.4 | 0.844(0.323-2.202) |
| P for trend |  |  | **0.019** |  |  |  | 0.428 |  |  |  | 0.759 |  |  |  | 0.477 |
| VAT |  |  |  |  |  |  |  |  |  |  |  |  |  |  |  |
| Q1 | 22 | 17.2 |  |  | 20 | 13.4 |  |  | 7 | 5.5 |  |  | 21 | 14.1 |  |
| Q2 | 33 | 37.5 | **3.934(1.876-8.249)** |  | 30 | 20.3 | 1.313(0.690-2.500) |  | 11 | 12.5 | 2.105(0.723-6.127) |  | 19 | 12.8 | 0.879(0.438-1.766) |
| Q3 | 28 | 62.2 | **11.183(4.506-27.754)** |  | 38 | 31.9 | **2.595(1.359-4.958)** |  | 11 | 24.4 | **4.550(1.444-14.337)** |  | 23 | 19.3 | 1.399(0.698-2.806) |
| Q4 | 15 | 68.2 | **16.923(5.218-54.886)** |  | 18 | 39.1 | **3.000(1.299-6.927)** |  | 9 | 40.9 | **8.974(2.440-33.011)** |  | 19 | 41.3 | **4.010(1.712-9.391)** |
| P for trend |  |  | **<0.001** |  |  |  | **0.001** |  |  |  | **<0.001** |  |  |  | **0.005** |
| BMI≥24kg/m2 |  |  |  |  |  |  |  |  |  |  |  |  |  |  |  |
| SAT |  |  |  |  |  |  |  |  |  |  |  |  |  |  |  |
| Q1 | 7 | 26.9 |  |  | 5 | 14.3 |  |  | 4 | 15.4 |  |  | 5 | 14.3 |  |
| Q2 | 30 | 50.0 | 2.046(0.696-6.010) |  | 14 | 43.8 | 2.417(0.658-8.881) |  | 11 | 18.3 | 1.203(0.320-4.522) |  | 13 | 40.6 | 2.170(0.588-8.011) |
| Q3 | 53 | 49.1 | 1.717(0.609-4.843) |  | 29 | 33.0 | 1.448(0.446-4.697) |  | 21 | 19.4 | 1.371(0.385-4.888) |  | 24 | 27.3 | 1.234(0.372-4.090) |
| Q4 | 74 | 51.0 | 1.503(0.511-4.422) |  | 43 | 28.9 | 0.790(0.233-2.671) |  | 37 | 25.5 | 1.818(0.484-6.824) |  | 30 | 20.1 | 0.752(0.209-2.698) |
| P for trend |  |  | 0.853 |  |  |  | 0.061 |  |  |  | 0.232 |  |  |  | 0.081 |
| VAT |  |  |  |  |  |  |  |  |  |  |  |  |  |  |  |
| Q1 | 7 | 25.9 |  |  | 3 | 7.1 |  |  | 3 | 11.1 |  |  | 3 | 7.1 |  |
| Q2 | 26 | 38.2 | 1.241(0.425-3.625) |  | 10 | 22.7 | 2.508(0.608-10.343) |  | 10 | 14.7 | 1.597(0.366-6.967) |  | 17 | 38.6 | **7.229(1.835-28.481)** |
| Q3 | 52 | 46.8 | 1.728(0.604-4.943) |  | 22 | 30.1 | 3.141(0.806-12.233) |  | 26 | 23.4 | 3.078(0.743-12.760) |  | 17 | 23.3 | 3.632(0.909-14.514) |
| Q4 | 79 | 59.4 | **2.958(1.020-8.577)** |  | 56 | 38.6 | **4.085(1.057-15.794)** |  | 34 | 25.6 | 3.753(0.890-15.829) |  | 35 | 24.1 | 3.927(0.983-15.681) |
| P for trend |  |  | **0.003** |  |  |  | **0.036** |  |  |  | **0.017** |  |  |  | 0.551 |
| Data are presented as medians (inter-quartile ranges) or n (%) or OR (95%CI). BMI, body mass index;TG, total triglyceride; HDL-C, high density lipoprotein cholesterol; The ORs was adjusted for age, BMI (for overall), smoke, drink, and menstrual history (for female). Male: SAT :Q1(＜98.1), Q2(98.1-), Q3(123.2-),Q4(149.8-);VAT:Q1(＜55.1), Q2(55.1-), Q3(91.00-), Q4(127.4-);Female: SAT:Q1(＜139.1), Q2(139.1-),Q3(178.2-),Q4(221.6-); VAT:Q1(＜43.0), Q2(43.0-), Q3(60.4-), Q4(79.6-). | | | | | | | | | | | | | | | |

| Supplemental Table 2. The relationships between levels of SAT, VAT and high-BS, high-BP stratified by sex and BMI | | | | | | | | | | | | | | | |
| --- | --- | --- | --- | --- | --- | --- | --- | --- | --- | --- | --- | --- | --- | --- | --- |
|  | High-BS(male) | | |  | High-BS(female) | | |  | High-BP(male) | | |  | High-BP(female) | | |
|  | n | % | OR (95%CI) |  | n | % | OR (95%CI) |  | n | % | OR (95%CI) |  | n | % | OR (95%CI) |
| Overall |  |  |  |  |  |  |  |  |  |  |  |  |  |  |  |
| SAT |  |  |  |  |  |  |  |  |  |  |  |  |  |  |  |
| Q1 | 21 | 13.5 |  |  | 34 | 17.8 |  |  | 69 | 44.8 |  |  | 46 | 24.1 |  |
| Q2 | 38 | 24.5 | 1.657(0.877-3.132) |  | 31 | 16.2 | 0.672(0.379-1.192) |  | 94 | 60.6 | 1.421(0.853-2.366) |  | 63 | 33.0 | 1.143(0.694-1.883) |
| Q3 | 36 | 23.2 | 1.348(0.671-2.709) |  | 25 | 13.0 | 0.423(0.223-0.800) |  | 97 | 62.6 | 1.306(0.739-2.308) |  | 57 | 29.5 | 0.697(0.408-1.192) |
| Q4 | 33 | 21.3 | 0.958(0.412-2.229) |  | 35 | 18.3 | **0.383(0.185-0.792)** |  | 105 | 67.7 | 1.373(0.698-2.701) |  | 91 | 47.6 | 0.790(0.430-1.449) |
| *P* for trend |  |  | 0.661 |  |  |  | **0.005** |  |  |  | 0.468 |  |  |  | 0.202 |
| VAT |  |  |  |  |  |  |  |  |  |  |  |  |  |  |  |
| Q1 | 21 | 13.5 |  |  | 19 | 9.9 |  |  | 63 | 40.9 |  |  | 38 | 19.9 |  |
| Q2 | 32 | 20.5 | 1.494(0.780-2.859) |  | 29 | 15.1 | 1.350(0.711-2.562) |  | 88 | 56.4 | 1.623(0.980-2.687) |  | 52 | 27.1 | 1.213(0.723-2.036) |
| Q3 | 38 | 24.4 | 1.676(0.836-3.359) |  | 29 | 15.1 | 1.212(0.624-2.351) |  | 95 | 60.9 | 1.742(0.990-3.065) |  | 58 | 30.2 | 1.098(0.643-1.874) |
| Q4 | 37 | 23.9 | 1.487(0.697-3.170) |  | 48 | 25.1 | 2.029(0.984-4.183) |  | 119 | 76.8 | **3.485(1.841-6.599)** |  | 109 | 57.1 | **2.211(1.234-3.961)** |
| *P* for trend |  |  | 0.305 |  |  |  | 0.055 |  |  |  | **<0.001** |  |  |  | **0.004** |
| BMI＜24kg/m2 |  |  |  |  |  |  |  |  |  |  |  |  |  |  |  |
| SAT |  |  |  |  |  |  |  |  |  |  |  |  |  |  |  |
| Q1 | 20 | 15.5 |  |  | 27 | 17.3 |  |  | 55 | 43.0 |  |  | 34 | 21.8 |  |
| Q2 | 21 | 22.1 | 1.623(0.729-3.613) |  | 21 | 13.2 | 0.519(0.263-1.026) |  | 58 | 61.1 | 1.483(0.772-2.851) |  | 49 | 30.8 | 1.234(0.705-2.161) |
| Q3 | 10 | 21.3 | 1.631(0.583-4.560) |  | 11 | 10.5 | **0.354(0.150-0.836)** |  | 25 | 53.2 | 0.865(0.375-1.994) |  | 24 | 22.9 | 0.769(0.392-1.506) |
| Q4 | 2 | 20.0 | 1.793(0.321-10.015) |  | 6 | 14.3 | 0.357(0.117-1.086) |  | 3 | 30.0 | 0.456(0.102-2.047) |  | 15 | 35.7 | 1.010(0.426-2.392) |
| *P* for trend |  |  | 0.306 |  |  |  | **0.017** |  |  |  | 0.447 |  |  |  | 0.61 |
| VAT |  |  |  |  |  |  |  |  |  |  |  |  |  |  |  |
| Q1 | 18 | 14.1 |  |  | 14 | 9.4 |  |  | 49 | 38.6 |  |  | 26 | 17.4 |  |
| Q2 | 18 | 20.5 | 1.671(0.748-3.735) |  | 22 | 14.9 | 1.395(0.657-2.962) |  | 52 | 59.1 | 1.755(0.929-3.314) |  | 43 | 29.1 | 1.663(0.916-3.019) |
| Q3 | 14 | 31.1 | **2.946(1.156-7.511)** |  | 22 | 18.5 | 1.762(0.816-3.806) |  | 27 | 60.0 | 1.594(0.717-3.541) |  | 28 | 23.5 | 1.138(0.589-2.198) |
| Q4 | 3 | 13.6 | 1.045(0.249-4.380) |  | 7 | 15.2 | 1.283(0.440-3.743) |  | 13 | 59.1 | 1.636(0.575-4.658) |  | 25 | 54.3 | **3.827(1.699-8.624)** |
| *P* for trend |  |  | 0.209 |  |  |  | 0.326 |  |  |  | 0.222 |  |  |  | **0.024** |
| BMI≥24kg/m^2^ |  |  |  |  |  |  |  |  |  |  |  |  |  |  |  |
| SAT |  |  |  |  |  |  |  |  |  |  |  |  |  |  |  |
| Q1 | 1 | 3.8 |  |  | 7 | 20 |  |  | 14 | 53.8 |  |  | 12 | 34.3 |  |
| Q2 | 17 | 28.3 | 6.767(0.804-56.963) |  | 10 | 31.3 | 1.334(0.369-4.822) |  | 36 | 60.0 | 1.098(0.394-3.062) |  | 14 | 43.8 | 0.732(0.208-2.584) |
| Q3 | 26 | 24.1 | 5.193(0.635-42.480) |  | 14 | 15.9 | 0.538(0.165-1.750) |  | 72 | 66.7 | 1.431(0.530-3.867) |  | 33 | 37.5 | 0.551(0.179-1.690) |
| Q4 | 31 | 21.4 | 2.863(0.335-24.431) |  | 29 | 19.5 | 0.522(0.155-1.757) |  | 102 | 70.3 | 1.622(0.566-4.653) |  | 76 | 51.0 | 0.591(0.187-1.863) |
| *P* for trend |  |  | 0.313 |  |  |  | 0.085 |  |  |  | 0.223 |  |  |  | 0.415 |
| VAT |  |  |  |  |  |  |  |  |  |  |  |  |  |  |  |
| Q1 | 3 | 11.1 |  |  | 5 | 11.9 |  |  | 14 | 51.9 |  |  | 12 | 28.6 |  |
| Q2 | 14 | 20.6 | 1.515(0.365-6.284) |  | 7 | 15.9 | 1.183(0.312-4.487) |  | 36 | 52.9 | 0.929(0.342-2.523) |  | 9 | 20.5 | 0.377(0.120-1.185) |
| Q3 | 24 | 21.6 | 1.405(0.349-5.650) |  | 7 | 9.6 | 0.529(0.134-2.092) |  | 68 | 61.3 | 1.278(0.473-3.448) |  | 30 | 41.1 | 0.697(0.249-1.946) |
| Q4 | 34 | 25.6 | 1.527(0.376-6.200) |  | 41 | 28.3 | 1.885(0.536-6.627) |  | 106 | 79.7 | **3.118(1.105-8.799)** |  | 84 | 57.9 | 1.020(0.372-2.795) |
| *P* for trend |  |  | **0.714** |  |  |  | 0.115 |  |  |  | **0.001** |  |  |  | 0.173 |
| Data are presented as medians (inter-quartile ranges) or n (%) or OR (95%CI). BMI, body mass index. High-BS: high blood sugar; High-BP: high blood pressure; The ORs was adjusted for age, BMI (for overall), smoke, drink, and menstrual history (for female). Male: SAT :Q1(＜98.1), Q2(98.1-), Q3(123.2-),Q4(149.8-);VAT:Q1(＜55.1), Q2(55.1-), Q3(91.00-), Q4(127.4-);Female:SAT:Q1(＜139.1), Q2(139.1-),Q3(178.2-),Q4(221.6-); VAT:Q1(＜43.0), Q2(43.0-), Q3(60.4-), Q4(79.6-). | | | | | | | | | | | | | | | |

| Supplemental Table 3. The associations of Z-score-transformed SAT and VAT with the metabolic abnormalities stratified by sex | | | | | | | | | |
| --- | --- | --- | --- | --- | --- | --- | --- | --- | --- |
|  | Male | | | |  | Female | | | |
|  | MV | *P* | MV +BMI | *P* |  | MV | *P* | MV +BMI | *P* |
| SAT |  |  |  |  |  |  |  |  |  |
| High TG | 1.40(1.18, 1.65) | **<0.001** | 1.03(0.80, 1.33) | 0.799 |  | 1.17(0.99, 1.38) | 0.063 | 0.90(0.70, 1.14) | 0.365 |
| Low HDL-C | 1.42(1.15, 1.74) | **0.001** | 1.20(0.88, 1.62) | 0.251 |  | 1.16(0.97, 1.40) | 0.107 | 0.85(0.66, 1.11) | 0.238 |
| High-BS | 1.20(0.99,1.46) | 0.068 | 1.08(0.79,1.46) | 0.638 |  | 0.94(0.77,1.15) | 0.543 | 0.72(0.55,0.96) | **0.025** |
| High-BP | 1.44(1.21,1.72) | **<0.001** | 1.19(0.91,1.55) | 0.202 |  | 1.35(1.15,1.59) | **<0.001** | 0.77(0.61,0.98) | **0.035** |
| MA | 1.66(1.38,1.99) | **<0.001** | 1.15(0.88,1.50) | 0.307 |  | 1.38(1.17,1.61) | **<0.001** | 0.76(0.60,0.96) | **0.023** |
| VAT |  |  |  |  |  |  |  |  |  |
| High TG | 2.07(1.72, 2.50) | **<0.001** | 2.16(1.69, 2.76) | **<0.001** |  | 1.53(1.29, 1.81) | **<0.001** | 1.47(1.19, 1.83) | **<0.001** |
| Low HDL-C | 1.81(1.46, 2.25) | **<0.001** | 1.83(1.38, 2.42) | **<0.001** |  | 1.55(1.29, 1.86) | **<0.001** | 1.47(1.17, 1.85) | **0.001** |
| High-BS | 1.26(1.03,1.53) | **0.024** | 1.18(0.90,1.54) | 0.230 |  | 1.27(1.05,1.53) | **0.014** | 1.28(1.01,1.64) | **0.044** |
| High-BP | 1.76(1.46,2.13) | **<0.001** | 1.71(1.33,2.19) | **<0.001** |  | 1.80(1.51,2.14) | **<0.001** | 1.41(1.14,1.74) | **0.002** |
| MA | 2.50(2.03,3.07) | **<0.001** | 2.42(1.85,3.17) | **<0.001** |  | 2.17(1.80,2.62) | **<0.001** | 1.77(1.41,2.22) | **<0.001** |
| Data was presented as OR (95%CI). MV: adjusted for age, smoke, drink, and menstrual history (for female); SAT, subcutaneous adipose tissue; VAT, visceral adipose tissue; MA: Metabolic abnormality; T2D, type 2 diabetes. OR, odds ratio; CI, confidence interval. | | | | | | | | | |
